# A new enzyme-linked immunosorbent assay for neurofilament light in cerebrospinal fluid: analytical validation and clinical evaluation

**DOI:** 10.1186/s13195-018-0339-1

**Published:** 2018-01-23

**Authors:** Lorenzo Gaetani, Kina Höglund, Lucilla Parnetti, Fani Pujol-Calderon, Bruno Becker, Paolo Eusebi, Paola Sarchielli, Paolo Calabresi, Massimiliano Di Filippo, Henrik Zetterberg, Kaj Blennow

**Affiliations:** 1 Section of Neurology, Department of Medicine, Santa Maria della Misericordia Hospital, University of Perugia, Perugia, Italy; 2 Institute of Neuroscience and Physiology, Department of Psychiatry and Neurochemistry, The Sahlgrenska Academy at the University of Gothenburg, Mölndal, Sweden; 3 Clinical Neurochemistry Laboratory, Sahlgrenska University Hospital, Mölndal, Sweden; 4 IRCCS Fondazione Santa Lucia, Rome, Italy; 5 Department of Molecular Neuroscience, UCL Institute of Neurology, Queen Square, London, UK; 6 UK Dementia Research Institute at UCL, London, UK

**Keywords:** Neurofilament light, ELISA, Cerebrospinal fluid, Multiple sclerosis, Clinically isolated syndrome, Alzheimer’s disease, Mild cognitive impairment, Parkinson’s disease

## Abstract

**Background:**

Cerebrospinal fluid (CSF) neurofilament light (NfL) is a reliable marker of neuro-axonal damage in different neurological disorders that is related to disease severity. To date, all recent studies performed in human CSF have used the same enzyme-linked immunosorbent assay (ELISA). To confirm the large body of evidence for NfL, we developed a new ELISA method and here we present the performance characteristics of this new ELISA for CSF NfL in different neurological disorders.

**Methods:**

We produced two monoclonal antibodies (NfL21 and NfL23) directed against the NfL core domain, and developed a novel sandwich ELISA method that we evaluated in patients with: 1) inflammatory demyelinating diseases (IDD; *n* = 97), including multiple sclerosis (MS; *n* = 59), clinically isolated syndrome (CIS; *n* = 32), and radiologically isolated syndrome (RIS; *n* = 6); 2) Alzheimer’s disease (AD; *n* = 72), including mild cognitive impairment due to AD (MCI-AD, *n* = 36) and probable AD dementia (AD-dem; *n* = 36); 3) Parkinson’s disease (PD; *n* = 30); and 4) other neurological noninflammatory and non-neurodegenerative diseases (OND; *n* = 30).

**Results:**

Our new NfL ELISA showed a good analytical performance (inter-plate coefficient of variation (CV) < 13%), with no cross-reactivity with neurofilament medium and heavy (NfM and NfH). With respect to the other available ELISAs, CSF NfL showed the same range of values with a strong correlation (*r* = 0.9984, *p* < 0.001) between the two methods. CSF NfL levels were significantly higher in MCI-AD/AD-dem and IDD patients as compared with both PD and OND patients. The highest discriminative power was obtained between IDD and OND patients (area under the curve (AUC) 0.87, 95% confidence interval (CI) 0.80–0.95). Within the IDD group, CSF NfL positively correlated with several clinical and radiological disease severity parameters.

**Conclusions:**

These results show a good analytical performance of the new ELISA for quantification of NfL concentrations in the CSF. CSF NfL is confirmed to be a reliable marker in AD and MS, and a disease-severity marker in MS patients.

## Background

In the personalized medicine era, management of diseases requires validated biomarkers to address the issues of making a clinical diagnosis, coming to a prognosis, and monitoring the biochemical effects of pharmacological treatments [1]. Cerebrospinal fluid (CSF) is a fluid of high value for biomarker discovery in neurological diseases since it is located close to the brain parenchyma and may reflect the pathological processes taking place within the central nervous system (CNS) more precisely than other body fluids [2].

Neurofilaments (Nf) are intermediate filaments of the neuronal cytoskeleton [3]. There are four different subunits called alpha-internexin, neurofilament light (NfL), medium (NfM), and heavy (NfH). Among them, NfL has a molecular weight of 68 kDa, is highly expressed in large-caliber myelinated axons, and has structural functions since it confers tensile strength to axons and dendrites [3, 4].

Low amounts of NfL are physiologically secreted into the CSF, but in patients with CNS pathology associated with axonal injury or degeneration, increased amounts of NfL are released from neurons into the interstitial space and into the CSF [3]. Because of the widespread localization of NfL within the CNS, an increase in its concentration in the CSF is considered a general marker of neuro-axonal damage [5]. Increased CSF NfL concentrations have been reported in multiple sclerosis (MS) [6], subcortical vascular dementia [7], Alzheimer’s disease (AD) [5], frontotemporal dementia (FTD) [8], Creutzfeldt-Jakob disease (CJD) [9], atypical parkinsonisms [10], normal pressure hydrocephalus [11], amyotrophic lateral sclerosis (ALS) [12], CNS infections [13], and brain traumatic injury [14]. CSF NfL is a promising marker for identifying neurodegenerative diseases, reliably measuring the degree of the ongoing neuro-axonal damage, and defining the prognosis of several neurological diseases and the response to treatment with disease-modifying drugs [6].

Reproducibility of the measurements with different assays is mandatory for the use of a biomarker in a clinical setting [15]. Rosengren and colleagues developed the first NfL enzyme-linked immunosorbent assay (ELISA) in 1996 [16]. This assay was based on polyclonal antisera. A few years later, monoclonal antibodies against NfL were developed [17], and a new NfL ELISA was established [18]. Since then, all the studies performed on NfL with an ELISA have used the same sandwich method (NF-light® ELISA kit; UmanDiagnostics AB, Umeå, Sweden).

To broaden the available choices of NfL measurement methods, we have generated novel monoclonal antibodies (NfL21 and NfL23) specific for NfL and established a new ELISA for the quantification of NfL in CSF. In this paper, we present the development and performance characteristics of this method. Moreover, we have applied this new assay to quantify NfL in the CSF of patients with a variety of neurological disorders, including inflammatory demyelinating diseases (IDD) of the CNS, AD, including both mild cognitive impairment due to AD (MCI-AD) and probable AD dementia (AD-dem), Parkinson’s disease (PD) and other neurological noninflammatory and non-neurodegenerative diseases (OND).

## Methods

### Assay development

#### Expression of human recombinant NfL

NfL-head + core, amino acid 1–396 of NfL, NfL-core, amino acid 93–396, and NfL-tail, amino acid 397–543, were amplified through a polymerase chain reaction (PCR) using primers containing 5′ *Bacillus amyloliquefaciens H* restriction enzyme I (BamHI) and 3′ *Escherichia coli* restriction enzyme I (EcoRI) sites, with full length complementary DNA (cDNA) for NfL (RC205920, Origene) as a template. The PCR fragments were purified and cloned into BamHI/EcoRI digested pGEX2T, a glutathione-S-transferase (GST) expression plasmid (GE Healthcare). Constructs were sequenced and transformed into *E. coli* BL21 (DE3). *E. coli* BL21(DE3) containing the different constructs was incubated overnight in 50 ml of lysogeny broth (LB) medium with ampicillin at a concentration of 100 μg/ml. The overnight culture was used to inoculate 1 liter of LB medium with ampicillin (100 μg/ml), and when the optical density measured at a wavelength of 600 nm (OD_600_) reached 0.4–0.6, protein expression was induced with 0.5 mM isopropyl β-D-1-thiogalactopyranoside (IPTG) for 4 h at +30 °C. The culture was centrifuged at 6000 rpm for 20 min at +4 °C and the pellet weight was determined. The pellet was stored at –20 °C pending purification. The pellet was re-suspended in 5 ml/mg of lysis buffer (20 mM Tris, 150 mM NaCl, 1% NP40 pH 7.5) plus complete protease inhibitors (Complete, Roche) and incubated with rotation for 30 min at room temperature, after which the lysate was centrifuged at 12,000 rpm for 20 min at +4 °C and the supernatant was collected. Protein extract was added to 50% Glutathione-Sepharose 4B (GE Healthcare) equilibrated with phosphate-buffered saline (PBS) and incubated for 30 min with rotation at room temperature. Sepharose was washed with PBS and the GST-NfL fusion protein was incubated with elution buffer (100 mM TrisHCl, 120 mM NaCl pH 8.0, with 20 mM glutathione) for 10 min. The unbound fusion protein was eluted and the incubation step was repeated four times. On-bead cleavage of the GST-fusion protein by thrombin was performed in PBS with 50 U thrombin for 2.5 h at room temperature. The cleaved, untagged protein was eluted with PBS containing protease inhibitor.

#### Characterization of specific NfL monoclonal antibodies

Monoclonal antibodies against NfL were generated by immunization of 8-week-old Balb/c mice with the recombinant protein fragments (head + core or core) in complete Freund’s adjuvant (Sigma). After 2–3 dosages with the recombinant protein fragment (approximately 75 μg/mouse), the spleen was removed and B cells were fused with the myeloma cell line SP2/0 following standard procedures. Approximately 10 days after fusion, cell media were screened for NfL antibodies using full-length recombinant NfL, recombinant protein fragment head + core (produced as described above), and purified bovine NfL protein [19]. Clones that reacted with the recombinant NfL proteins and bovine NfL, but not with negative control protein APLP1 (amyloid-like precursor protein 1) were further grown, subcloned, and subsequently frozen in liquid nitrogen. The isotype was determined using a commercially available kit (Pierce Rapid Isotyping Kit-Mouse). Finally, antibodies were purified using a protein G column (GE Healthcare).

#### Surface plasmon resonance and immunoprecipitation/Western blot analyses

Anti-mouse antibody (mouse antibody capture kit BR-1008-38; GE Healthcare) was immobilized according to the kit instructions on a C1 Biacore chip (BR-1005-40) in a Biacore X100 instrument via amine-coupling at 25 °C in running buffer (PBS with 5% DMSO and 0.05% Tween 20). A final response of 1100 RU for the immobilization level was obtained. This chip was then used at 37 °C to bind the NfL capture antibody to be analyzed (3 nM in running buffer with 1 mg/ml CM-Dextran (CMD); Sigma Aldrich 86524) and, subsequently, dilutions of bovine NfL (dilution series of two-fold dilutions in running buffer with 1 mg/ml CMD, from 30 nM NfL to 1.9 nM, and 0 nM; duplicate samples at the 15 nM NfL concentration). For regeneration between cycles of this multicycle measuring method, we used injection of 30 mM HCl for 2 min and of 10 mM glycine, pH 1.5, for 30 s (all at 10 μl/min), before repeating the cycle for the next NfL concentration. Antibody binding: contact time: 180 s, flow rate 10 μl/min, stabilization period 300 s; NfL antigen binding: contact time 180 s, flow rate 30 μl/min, dissociation time: 900 s. Kinetic analysis was performed using the 1:1 binding model of the Biacore X100 Evaluation software (V2.0.1).

For immunoprecipitation and Western blot, the NfL21 and NfL23 monoclonal antibodies were bound to magnetic Dynabeads M-280 Sheep Anti-Mouse IgG, and incubated with CSF samples, head, core and tail recombinant fragments of NfL or full-length NfL recombinant protein (Origene). After elution, bound proteins were electrophoresed on a NuPAGE 4–12% Bis-Tris gel and transferred to Amersham™ Protran™ Nitrocellulose membranes, probed with NfL21 or NfL23 using the ECL Select™ Western Blotting Detection Reagent (GE Healthcare).

### Selection and analysis of CSF samples

#### CSF sampling

We selected 235 CSF samples stored in the CSF Biobank of the Section of Neurology, Department of Medicine, University of Perugia (Perugia, Italy), for the study. CSF was collected over a 10-year period (2006–2016) via lumbar puncture at the same institution using the same standard operating procedures throughout the study following international guidelines [20]. Specifically, lumbar puncture was performed between 8:00 and 10:00 a.m. and CSF was collected in sterile polypropylene tubes, centrifuged for 10 min at 2000 × g, divided into 0.5-ml aliquots and immediately frozen at −80 °C pending analysis. After lumbar puncture, patient demographic and clinical data were stored in an online electronic database. The selected CSF samples belonged to four groups of patients: 1) inflammatory demyelinating diseases of the CNS (IDD group); 2) probable AD dementia and mild cognitive impairment (MCI) due to AD (MCI-AD/AD-dem group); 3) clinically established PD (PD group); and 4) patients with other neurological diseases (OND) (OND group). For patients belonging to IDD, MCI-AD/AD-dem, and OND groups, CSF was collected as part of their diagnostic work-up, whilst patients in the PD group underwent lumbar puncture for research purposes.

#### Selection of CSF samples

For the IDD group, we selected CSF samples from patients satisfying the following criteria at the time of CSF collection: 1) a diagnosis of clinically isolated syndrome suggestive of MS (CIS), relapsing-remitting MS (RRMS), primary progressive MS (PPMS) or secondary progressive MS (SPMS) according to the 2010 revision of the McDonald criteria [21], or a diagnosis of radiologically isolated syndrome (RIS) according to the criteria by Okuda et al. 2009 [22]; and 2) age > 18 years. The CSF samples of the MCI-AD/AD-dem group were selected from patients satisfying the following criteria at the time of CSF collection: 1) a diagnosis of MCI with impairment in episodic memory and with evidence of a progressive decline in cognitive performance over time (MCI-AD) or probable AD dementia (AD-dem) according to the recommendations from the National Institute on Aging—Alzheimer’s Association (NIA-AA) workgroups on diagnostic guidelines [23, 24]; and 2) age > 55 years. For the PD group, we selected CSF samples from patients satisfying the following criteria at the time of CSF collection: 1) a diagnosis of clinically established PD according to the Movement Disorder Society (MDS) clinical diagnostic criteria for PD [25]; and 2) age > 55 years. For all groups, the reference diagnostic criteria were retrospectively applied based on the medical records stored in our electronic database. Finally, the CSF samples for the OND group were selected from patients with a diagnosis other than inflammatory or degenerative disease of the CNS or of the peripheral nervous system and an age > 18 years at the time of the CSF collection.

#### IDD patients: clinical assessment

A senior neurologist expert in inflammatory demyelinating diseases of the CNS examined all the study participants and scored the Kurtzke’s Expanded Disability Status Scale (EDSS) [26]. The presence of CSF IgG oligoclonal bands (OCB) was routinely assessed with agarose gel isoelectrofocusing followed by immunoblotting, as recommended [27]. Patients were considered OCB-positive if having > 1 CSF OCB, and considered OCB-negative if having 0–1 CSF OCB [27]. At the time of the lumbar puncture, patients also underwent a 1.5 Tesla brain and spinal cord contrast-enhanced magnetic resonance imaging (MRI) [28]. A neuroradiologist examined all the MRI scan data of the enrolled patients. Patients were followed-up clinically and radiologically according to the routine clinical practice. Since in all CSF was collected during the diagnostic work-up, none of the patients were on disease-modifying therapy at the time of lumbar puncture.

#### MCI-AD/AD-dem patients: clinical assessment

A senior neurologist expert in AD and memory disturbances examined all the study participants. A neuropsychologist performed neuropsychological evaluations, including Mini Mental Status Examination (MMSE) [29], at the time of CSF collection and then during the follow-up.

#### PD patients: clinical assessment

A senior neurologist expert in movement disorders examined all the patients and performed extensive clinical testing, including the scoring of the Unified Parkinson’s Disease Rating Scale (UPDRS) part III [30] and of the Hoehn & Yahr (H&Y) scale [31]. Neuropsychological evaluations were performed by a neuropsychologist at the baseline and during the follow-up.

#### NfL ELISA

NfL21 was used as capturing antibody and was coated on Nunc maxisorp 96-well microtiter plates at a final concentration of 0.5 μg/ml (100 μl/well) in 50 mM bicarbonate buffer, pH 9.6, overnight at +4 °C. All washes were performed at room temperature by adding 4 × 350 μl PBS containing 0.05% Tween20 (PBS-T). After a first wash, the remaining protein binding sites were blocked at room temperature with 1% bovine serum albumin (BSA) in PBS (0.01 M phosphate buffer, 0.14 M NaCl, pH 7.4) for 1 h (250 μl/well). Thereafter, plates were washed and 100 μl of calibrator (range 39–5000 pg/ml), blank, quality-control (QC) samples, and CSF were added to the corresponding wells. QC samples and CSF were diluted 1:2 in PBS-T. The plate was shaken for 1 h at 400 rpm and then incubated overnight at +4 °C. After washing, 100 μl of biotinylated NfL23 detection antibody (0.4 μg/ml in PBS-T) was added and incubated for 2.5 h at room temperature. After washing, enhanced streptavidin horseradish peroxidase (Enhanced Streptavidin-HRP, 4740 N, Kem En Tech,) diluted 1:20,000 in PBST 0.05% BSA 1% was added (100 μl/well), and incubated for 30 min at room temperature. After a final wash, 100 μl 3,3′,5,5′-tetramethylbenzidine (TMB) substrate (TMB One Solution, 4380A, Kem En Tech) was added to generate the color. After 20-min incubation in the dark at room temperature the reaction was stopped by the addition of 100 μl 2 M H_2_SO_4_ and the absorbance was measured at 450 nm (reference wavelength 650 nm) using an ELISA plate reader (Vmax, Molecular Devices, USA). A fitted four-parameter logistic model was used to generate the calibration curve and the blank was included as zero concentration of NfL. Validation was performed by evaluating the assay range, the upper and lower limit of quantification (ULOQ, LLOQ), within and inter-plate precision, linear dilution, cross-reactivity, and spike recovery during five separate analyses on different days. Cross-reactivity against NfM and NfH was evaluated by spiking in NfM or NfH into the calibrator curve. Spike recovery was performed by spiking in bovine NfL into five individual CSF samples with endogenous NfL levels ranged between 1208–6649 pg/ml. Recovery was calculated by subtracting the measured concentration of the unspiked samples from the measured concentration of the spiked sample, divided by the spike concentration.

### Statistical analysis

A likelihood ratio test based on generalized linear models was used to test if the distribution of demographics, clinical, and biochemical features differed between IDD subgroups and between MCI-AD/AD-dem and PD groups. The procedure was carried out to control for the age distribution. Logarithmic transformation was applied to NfL values to reach normality, as verified with the Shapiro-Wilk test. To evaluate potential confounders, we tested the association between CSF NfL logarithmic (log) values and age and gender. The association between CSF NfL log values and gender was tested with Student’s *t* test. To control for the confounding effect of age and gender, we carried out an analysis of covariance (ANCOVA) to verify if CSF NfL log values would differ between the diagnostic groups. Whenever appropriate, we considered age and gender as potential confounders in the analyses performed in each diagnostic group. The optimal diagnostic accuracy of NfL was assessed by calculating the area under the curve (AUC) of the receiver operating characteristic (ROC) curve at the point that maximized the Youden index. ROC analysis was performed both adjusting and not adjusting for age. All tests were two-sided and significance was set at *p* < 0.05. Statistical analyses were performed using R software, version 3.3.1.

## Results

### Characterization of monoclonal antibody NfL21 and NfL23

We used surface plasmon resonance (SPR) measurements to determine the kinetics of binding of bovine NfL to the NfL antibodies captured on a Biacore C1 chip. Both antibodies showed high affinity binding to bovine NfL (dissociation constant or K_D_ of NfL21, 0.3 nM; K_D_ of NfL23, 0.5 nM; the association rate constants ka (1/Ms) were similar for both NfL21 and NfL23 (around 4–5 × E05), and the dissociation rate constants (1/s) were also very similar for the two antibodies (around 1–2 × E-04); corresponding half-life of the antibody NfL complex between 58 and 115 min). The residuals for the curve fitting were within the acceptable range; however, NfL showed at the highest used concentration some background binding to the C1 chip even in absence of capture antibody which could not be improved by testing various regeneration conditions. The above given kinetic data must therefore be seen as preliminary estimates only. Immunoprecipitation and Western blot analysis showed that both the NfL21 and NfL23 antibodies recognized a band corresponding to full-length NfL, and reacted with the core domain, but not the head or core domains, of recombinant NfL (data not shown).

### Analytical performance of the NfL ELISA

**Fig. 1:**
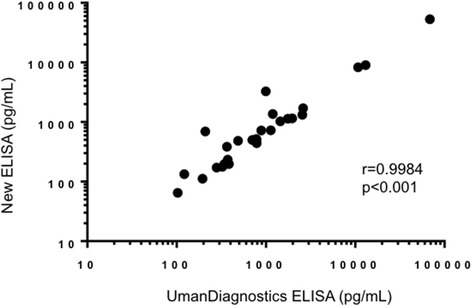
Scatter plots showing the correlation between CSF NfL values as measured with the new enzyme-linked immunosorbent assay (ELISA) and with the ELISA from UmanDiagnostics

The validation of the novel NfL ELISA confirmed an assay range between 39 pg/ml and 5000 pg/ml. Taking into account the two-fold dilution of samples, the LLOQ is 78 pg/ml and the ULOQ is 10,000 pg/ml. Furthermore, within-plate and inter-plate variations were below 8% and 13%, respectively. Samples diluted in a linear manner and spike recovery was between 80% and 109%. There was no cross-reactivity towards NfM or NfH. There was a strong correlation (*r* = 0.9984, *p* < 0.001; Fig. 1) between CSF NfL values analyzed using UmanDiagnostics ELISA and our new ELISA.

### Patient characteristics

The IDD group included 97 patients. Among them, 6 patients had a diagnosis of RIS, 32 of CIS, 51 of RRMS, and 8 of progressive MS (PMS). In this latter group, 2 patients were diagnosed with SPMS and 6 with PPMS. Patients with PMS had significantly higher mean age, disease duration, EDSS scores, and total number of T2 MRI lesions as compared to the other IDD subgroups. On the contrary, CIS and RRMS patients more frequently had clinical and neuroradiological signs of disease activity (both a recent relapse and a higher number of gadolinium enhancing (Gd+) lesions on MRI). The details of IDD patient characteristics are reported in Table 1. The MCI-AD/AD-dem group included 36 patients with MCI-AD and 36 patients with AD-dem. The PD group was composed of 30 patients with clinically established PD. MCI-AD/AD-dem patients had a significantly higher age, a shorter disease duration, and lower MMSE scores as compared to PD patients. Clinical characteristics of both groups are reported in Table 2. Finally, the OND group included 36 patients as neurological controls. OND patient characteristics and their specific diagnoses are reported in Table 3.

**Table 1:** Main characteristics of IDD patients

	IDD group					Differences between groups (*p* values)
	Total (*n* = 97)	RIS (*n* = 6)	CIS (*n* = 32)	RRMS (*n* = 51)	PMS (*n* = 8)	
Age (years)	38.7 ± 11.1; 37 (21–69)	46 ± 5.9; 47 (37–54)	38.3 ± 11.5; 38 (21–65)	36.7 ± 9.9; 35.5 (22–69)	46.6 ± 14.3; 44.5 (29–66)	0.038
Female/male	2.5 (69/28)	2 (4/2)	1.7 (20/12)	3.3 (39/12)	3 (6/2)	0.624
Recent relapse^a^	64 (65.9%)	0	27 (84.4%)	36 (70.6%)	1 (12.5%)	< 0.001
Time from clinical onset (years)	2.7 ± 4.9; 0.3 (0.001–29)	–	0.3 ± 1; 0.04 (0–5)	3.8 ± 6; 1 (0.01–29)	5.1 ± 3.3; 4.5 (2–10)	0.010
EDSS	2.2 ± 1.3; 2 (0–7)	1.1 ± 0.6; 1 (0–2)	1.9 ± 0.8; 2 (1–4)	2.2 ± 1.2; 2 (0–7)	4.7 ± 1.7; 4.5 (3–7)	< 0.001
OCB+	68 (70.1%)	4 (66.7%)	19 (59.4%)	37 (72.5%)	8 (100%)	0.040
T2 lesions (*n*)	9.4 ± 7.2; 8 (1–39)	6.1 ± 3.6; 5 (3–11)	6.2 ± 4.7; 5 (1–18)	11.6 ± 6.3; 10 (2–36)	18.6 ± 11; 16 (7–39)	< 0.001
0–3 T2 lesions	21 (21.6%)	1 (16.7%)	11 (34.4%)	10 (19.6%)	0	< 0.001
4–9 T2 lesions	36 (37.1%)	3 (50%)	15 (46.9%)	15 (29.4%)	2 (25%)	
>9 T2 lesions	40 (41.2%)	2 (33.6%)	6 (18.7%)	26 (51%)	6 (75%)	
Juxtacortical lesions (*n*)	2.2 ± 2.1; 2 (0–9)	2 ± 1.2; 2 (0–3)	1.4 ± 1.8; 1 (0–6)	2.5 ± 2; 3 (0–9)	3.7 ± 3.5; 4 (0–9)	0.011
Periventricular lesions (*n*)	5 ± 3.2; 5 (0–15)	3.4 ± 2.6; 3 (0–7)	3.5 ± 3; 3 (0–10)	5.7 ± 2.7; 6 (0–12)	8.4 ± 3.8; 9 (2–15)	< 0.001
Infratentorial lesions (*n*)	1.1 ± 1.5; 0.5 (0–6)	0	0.5 ± 0.9; 0 (0–4)	1.4 ± 1.6; 1 (0–6)	2.6 ± 2.1; 2 (0–6)	< 0.001
Spinal cord lesions (*n*)	1.4 ± 2; 1 (0–9)	0.6 ± 1; 0 (0–2)	0.7 ± 1; 1 (0–5)	1.7 ± 2.1; 1 (0–9)	3.9 ± 3.1; 4 (0–9)	< 0.001
Gd + lesions (*n*)	1.4 ± 2.4; 1 (0–14)	0.3 ± 0.5; 0 (0–1)	0.6 ± 0.5; 1 (0–1)	2.2 ± 3; 1 (0–14)	0.1 ± 0.4; 0 (0–1)	0.004
0 Gd + lesions	43 (44.3%)	4 (66.7%)	12 (37.5%)	20 (39.2%)	7 (87.5%)	< 0.001
1–3 Gd + lesions	45 (46.4%)	2 (33.3%)	20 (62.5%)	22 (43.2%)	1 (12.5%)	
>3 Gd + lesions	9 (9.3%)	0	0	9 (17.6%)	0	
CSF NfL (pg/ml)	881 ± 941; 639 (68–5748)	438 ± 262; 321 (198–795)	791 ± 687; 631 (156–3492)	1028 ± 1157; 642 (136–5748)	1066 ± 904; 825 (350–3065)	0.345

Categorical variables are reported as numbers and percentages as compared to the reference group

Continuous variables are reported as means ± standard deviations; median (range)

*p* values are from likelihood ratio test based on generalized linear models and age-adjusted

*p* value for the comparison of CSF NfL between RIS, CIS, RRMS, and PMS groups is from ANCOVA

*CIS* clinically isolated syndrome suggestive of multiple sclerosis, *CSF* cerebrospinal fluid, *EDSS* Expanded Disability Status Scale, *Gd* + gadolinium-enhancing lesions, *NfL* neurofilament light, *OCB*+ evidence of CSF IgG oligoclonal bands, *PMS* progressive multiple sclerosis (both secondary and primary progressive multiple sclerosis), *RIS* radiologically isolated syndrome, *RRMS* relapsing remitting multiple sclerosis

^a^ Recent relapse: clinical episode of neurological deficit with onset in the 30 days preceding CSF collection

**Table 2:** Main characteristics of MCI-AD/AD-dem and PD patients

	MCI-AD/AD-dem group			PD group	Differences between groups *p* values
	Total (*n* = 72)	MCI-AD (*n* = 36)	AD-dem (*n* = 36)	Clinically established PD (*n* = 30)	
Age (years)	72 ± 5.6; 72 (58–83)	72.2 ± 5.6; 72.5 (62–82)	71.9 ± 6; 72 (58–83)	68.3 ± 7.5; 68 (55–86)	0.032
Female/male	1.4 (42/30)	1.25 (20/16)	1.6 (22/14)	1.5 (18/12)	0.797
Disease duration (years)	2.6 ± 1.9; 2 (0.2–7)	2.4 ± 1.4; 2 (0.5–5)	2.8 ± 1.9; 3 (0.2–7)	7 ± 9.4; 3 (0.5–35)	0.004
MMSE baseline	19.9 ± 4; 20.9 (3.2–30)	22.5 ± 4; 22.4 (10.2–30)	16.8 ± 5.3; 18.4 (3.2–25.3)	24.1 ± 4.6; 25.2 (13.2–30)	< 0.001
MMSE follow-up	17.4 ± 5.7; 17.5 (1.4–26.3)	19.1 ± 5.7; 18.7 (7.4–26.3)	14.8 ± 6.3; 15.3 (1.4–25)	22.9 ± 6; 25.3 (13.9–26.9)	0.043
ΔMMSE	4.9 ± 4.9; 3.7 (−5.9–16.6)	4.2 ± 4.9; 3.2 (−2.7–16.6)	6.3 ± 6.9; 6.6 (−5.9–16)	2.2 ± 3; 1.6 (−0.4–6.1)	0.346
Follow-up time (years)	2.3 ± 1.2; 2.1 (0.1–16.6)	4.2 ± 4.9; 3.2 (2.7–16.6)	2.2 ± 1.6; 2.1 (0.1–5.1)	1.4 ± 1.8; 0.6 (0.3–4)	0.439
H&Y	–	–	–	2 ± 1; 2 (1–4)	–
UPDRS III	–	–	–	28.2 ± 18.8; 25 (10.5–78)	–
CSF NfL (pg/ml)	1003 ± 484; 920 (391–3272)	906 ± 327; 919 (391–1936)	1099 ± 591; 923 (448–3272)	622 ± 461; 503 (171–2577)	0.003

Categorical variables are reported as numbers and percentages as compared to the reference group

Continuous variables are reported as means ± standard deviations; median (range)

*p* values are from likelihood ratio test based on generalized linear models and age-adjusted

*p* value for the comparison of CSF NfL between MCI-AD/AD-dem and PD groups is from ANCOVA (see Table 4)

*AD-dem* Alzheimer’s disease dementia, *CSF* cerebrospinal fluid, *H&Y* Hoen & Yahr scale, *MCI-AD* mild cognitive impairment due to Alzheimer’s disease, *PD* Parkinson’s disease, *MMSE* Mini Mental Status Examination (values adjusted for age and education), Δ*MMSE* difference between the MMSE score at the follow-up and the MMSE score at the baseline, *NfL* neurofilament light, *UPDRS* III Unified Parkinson’s Disease Rating Scale, part III

**Table 3:** Main characteristics of OND patients

	OND group Total (*n* = 36)
Age	56.3 ± 17.5; 59 (11–82)
Female/male	1 (18/18)
Specific diagnoses	
Headache	13 (36.1%)
Psychiatric disorders	10 (27.8%)
Idiopathic intracranial hypertension	5 (13.9%)
Drug induced parkinsonism	4 (11.1%)
Isolated cranial nerve palsy	2 (5.6%)
Medically unexplained neurological symptoms	2 (5.6%)
CSF NfL (pg/ml)	577 ± 548; 361 (120–2680)

Categorical variables are reported as numbers and percentages as compared to the reference group

Continuous variables are reported as means ± standard deviations; median (range)

*CSF* cerebrospinal fluid, *NfL* neurofilament light

### Impact of demographic characteristics on CSF NfL

For the entire cohort we found a positive correlation between CSF NfL concentrations and age (*r* = 0.19, *p* = 0.003). Moreover, NfL was slightly increased in males as compared to females (889 ± 625 pg/ml versus 808 ± 784 pg/ml, *p* = 0.036). For this reason, the differences in CSF NfL values between the diagnostic groups were tested with ANCOVA adjusted for age and gender. Furthermore, a positive correlation between CSF NfL concentrations and age was also found in the PD group (*r* = 0.38, *p* = 0.039). No such correlation was seen in the IDD or MCI-AD/AD-dem groups. There was no gender difference in CSF NfL concentration within the different diagnostic groups.

### Differences in CSF NfL between diagnostic groups

**Fig. 2:**
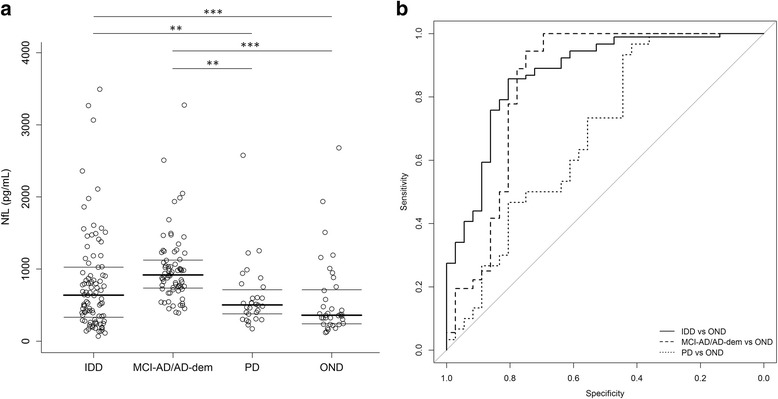
CSF NfL values (pg/ml) in the different diagnostic groups. **a** CSF neurofilament light (NfL) values in IDD, MCI-AD/AD-dem, PD, and OND groups; *p* values are from the ANCOVA adjusted for age and sex; *p < 0.05; **p < 0.01; ***p < 0.001. **b** Diagnostic value of CSF NfL. IDD vs OND comparison (age-adjusted AUC = 0.87, 95% CI 0.80–0.95) is reported as the solid line. MCI-AD/AD-dem vs OND comparison (age-adjusted AUC = 0.84, 95% CI 0.74–0.95) is reported as the dashed line. PD vs OND comparison (age-adjusted AUC = 0.69, 95% CI 0.56–0.81) is reported as the dotted line. *AD-dem* Alzheimer’s disease dementia, *IDD* inflammatory diseases of the central nervous system, *MCI-AD* mild cognitive impairment due to Alzheimer’s disease, *OND* other neurological diseases, *PD* Parkinson’s disease

CSF NfL values were significantly higher in both the IDD (881 ± 941 pg/ml) and the MCI-AD/AD-dem groups (1003 ± 484 pg/ml) as compared with the OND group (577 ± 548 pg/ml; *p* < 0.001 for both comparisons). Moreover, both IDD and MCI-AD/AD-dem patients had higher CSF NfL concentrations as compared with PD patients (622 ± 461 pg/ml; *p* = 0.032 and *p* = 0.003, respectively). No statistically significant difference was found between the IDD and MCI-AD/AD-dem groups or between the PD and OND groups (Table 4 and Fig. 2). ROC analysis was carried out to assess CSF NfL diagnostic value in distinguishing IDD, MCI-AD/AD-dem, and PD patients from the OND group. CSF NfL achieved the best diagnostic performance in discriminating IDD from OND patients (age-adjusted AUC = 0.87, 95% confidence interval (CI) 0.80–0.95) with a sensitivity of 0.86 (95% CI 0.74–0.9) and a specificity of 0.83 (95% CI 0.67–0.94), followed by MCI-AD/AD-dem versus OND patients (age-adjusted AUC = 0.84, 95% CI 0.74–0.95) with a sensitivity of 0.94 (95% CI 0.86–1.00) and a specificity of 0.75 (95% CI 0.61–0.89). Finally, CSF NfL discriminated PD from OND patients (age-adjusted AUC = 0.69, 95% CI 0.56–0.81) with a sensitivity of 0.97 (95% CI 0.90–1.00) and a specificity of 0.42 (95% CI 0.25–0.58) (Fig. 2). Unadjusted AUC values were 0.63 (95% CI 0.52–0.73) for the discrimination between IDD and OND patients, 0.83 (95% CI 0.72–0.93) for the discrimination between MCI-AD/AD-dem and OND patients, and 0.62 (95% CI 0.48–0.76) for the discrimination between PD and OND patients.

**Table 4:** Results of analysis of covariance comparing cerebrospinal fluid neurofilament light log values (pg/ml) in IDD, MCI-AD/AD-dem, PD, and OND patients

	Estimate	Standard error	*p* value
IDD vs. OND	0.64	0.16	< 0.001
MCI-AD/AD-dem vs. OND	0.64	0.15	< 0.001
PD vs. MCI-AD/AD-dem	−0.52	0.15	0.003
PD vs. IDD	−0.52	0.19	0.032
IDD vs. MCI-AD/AD-dem	0.12	0.17	0.894
PD vs. OND	0.00	0.18	1.000

The analysis has been adjusted for age and sex

*AD-dem* Alzheimer’s disease dementia, *IDD* inflammatory diseases of the central nervous system, *MCI-AD* mild cognitive impairment due to Alzheimer’s disease, *OND* other neurological diseases, *PD* Parkinson’s disease

### CSF NfL within the IDD group

**Fig. 3:**
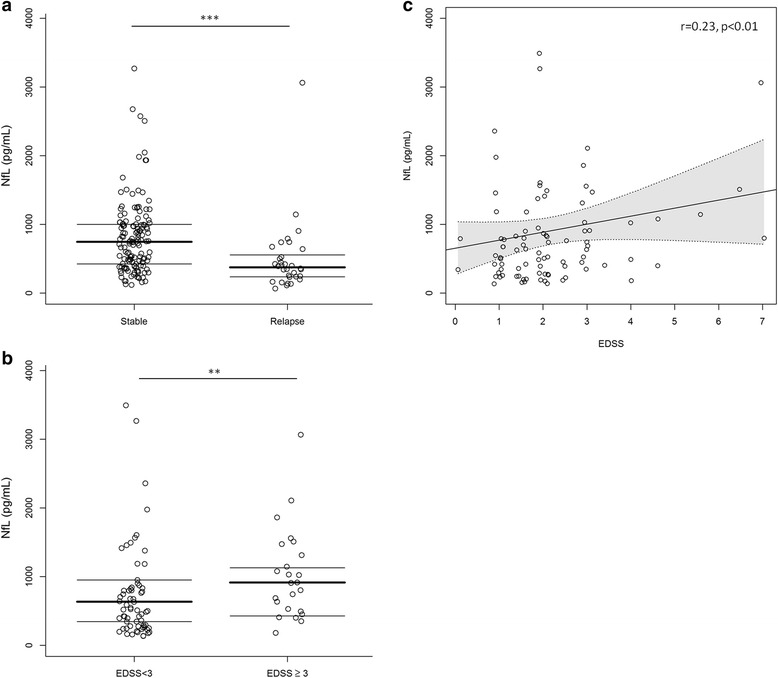
Associations between CSF NfL values (pg/ml) and clinical characteristics in IDD patients. **a** CSF neurofilament light (NfL) values in patients with and without a recent relapse (within 30 days prior to CSF collection) in the IDD group; ***p < 0.001. **b** Difference in CSF NfL values in patients with EDSS ≥ 3 and < 3; **p < 0.01. **c** Scatter plots showing the correlation between CSF NfL values and EDSS

Within the IDD group, CSF NfL concentrations were not significantly different in RIS, CIS, RRMS, and PMS patients. CSF NfL values, however, were significantly higher in patients with a recent relapse (defined as a clinical episode within 30 days prior to CSF collection) than in patients with no evidence of recent clinical disease activity (1073 ± 1041 pg/ml versus 509 ± 536 pg/ml, *p* < 0.001) (Fig. 3). Moreover, CSF NfL concentrations correlated with the degree of neurological impairment at the time of the lumbar puncture. Indeed, a positive correlation between CSF NfL values and EDSS scores was found (*r* = 0.23, *p* = 0.026). Furthermore, CSF NfL concentrations were significantly higher in patients with EDSS ≥ 3 than in patients with EDSS < 3 (1075 ± 1099 versus 799 ± 859 pg/ml, *p* = 0.007) (Fig. 3). No significant association was found between CSF NfL values and the presence of CSF OCB.

**Fig. 4:**
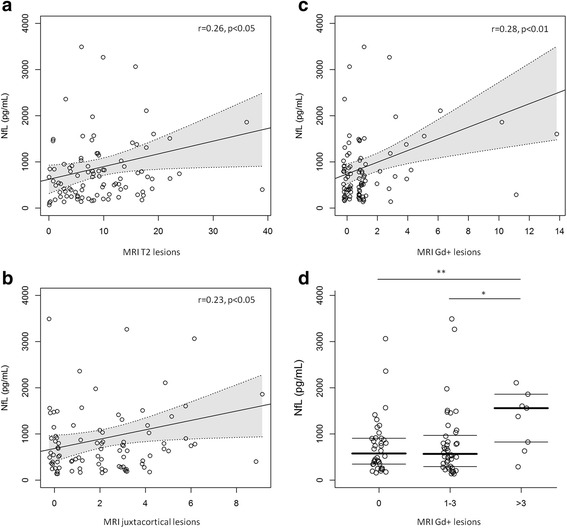
CSF NfL values (pg/ml) and magnetic resonance imaging (MRI) features in IDD patients. **a** Scatter plots showing the correlation between CSF neurofilament light (NfL) values and the total number of T2 lesions. **b** Scatter plots showing the correlation between CSF NfL values and the number of juxtacortical lesions. **c** Scatter plots showing the correlation between CSF NfL values and the total number of gadolinium enhancing (Gd+) lesions. **d** CSF NfL values in patients with 0 Gd + lesions, 1–3 Gd + lesions, and > 3 Gd + lesions. *p < 0.05, **p < 0.01

Several associations were found between CSF NfL values and MRI features. Specifically, CSF NfL concentrations correlated positively with the total number of T2 lesions (*r* = 0.26, *p* = 0.010) and the number of juxtacortical lesions (*r* = 0.23, *p* = 0.031), as well as with Gd+ lesions (*r* = 0.28, *p* = 0.008) at baseline MRI. CSF NfL values were also found to be significantly higher in patients with a higher number of Gd + lesions (*p* < 0.001). Post-hoc comparison showed that NfL values were increased in those patients with more than 3 Gd + lesions compared to 0 (*p* = 0.019) or 1–3 Gd + lesions in the baseline MRI (*p* = 0.025) (Fig. 4). Finally, CSF NfL values did not correlate with the time between the first clinical manifestation of the disease and the CSF collection.

### CSF NfL within the MCI-AD/AD-dem group

No statistically significant difference in CSF NfL concentrations was observed between patients with MCI-AD and patients with AD-dem. Moreover, no significant correlation was found between CSF NfL values and MMSE at baseline and follow-up. Likewise, the change of MMSE from the baseline to the follow-up did not show any significant correlation with CSF NfL concentrations. In addition, disease duration did not correlate with CSF NfL values.

### CSF NfL within the PD group

**Fig. 5:**
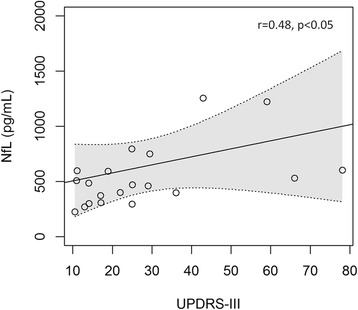
Scatter plots showing the correlation between CSF neurofilament light (NfL) values (pg/ml) and the Unified Parkinson’s Disease Rating Scale (UPDRS) III scores in PD patients

A statistically significant positive correlation between CSF NfL values and UPDRS III values was obtained with an unadjusted linear regression (*r* = 0.48, *p* = 0.028) (Fig. 5). However, after adjusting for age, the statistical significance disappeared. No statistically significant correlation was found between CSF NfL concentrations and H&Y scores, baseline and follow-up MMSE scores, or disease duration.

## Discussion

The primary finding of our study was the good analytical performance of the newly established ELISA for NfL. The assay demonstrates robust and accurate measurement of NfL in CSF (inter-plate coefficient of variation (CV) < 13%) with no cross-reactivity towards NfM or NfH. Absolute concentrations were in the same range as the ELISA from UmanDiagnostics and, importantly, there was a clear correlation. Further characterization of the antibodies revealed high affinity (subnanomolar K_D_ on Biacore) for NfL with epitopes in the core region of NfL. Given the obtained results, our assay represents a valid alternative for the measurement of NfL concentrations in the CSF.

The other main results from our study were: 1) a significant increase in CSF NfL in IDD and MCI-AD/AD-dem patients as compared with PD patients and to the control group; 2) no significant differences in CSF NfL in PD patients as compared with controls; 3) a significant association between CSF NfL and several clinical and radiological disease severity measures in IDD patients; and 4) no significant correlations between CSF NfL and clinical variables in MCI-AD/AD-dem and PD patients.

### CSF NfL in IDD patients

The increase of CSF NfL in IDD patients is in line with the results of several previous studies performed on this type of diseases showing that CSF NfL concentrations are significantly higher in CIS, RRMS, SPMS, and PPMS as compared with controls [32, 33]. Moreover, the diagnostic accuracy of our assay in distinguishing IDD patients from controls is similar to that previously reported with the other available ELISA. For instance, in the work by Kuhle and colleagues, the diagnostic accuracy of CSF NfL was calculated separately for CIS and MS patients and they found an AUC of 0.83 and 0.91, respectively [32]. In our study, we have considered RIS, CIS, and MS patients together within the IDD group, thus finding an age-adjusted AUC of CSF NfL of 0.87, in line with the above-mentioned previously reported AUC values. It should be noted that the unadjusted AUC for the discrimination between IDD patients and controls has been found to be smaller (0.63) than the age-adjusted AUC. This discrepancy could be due to the higher mean age of our control patients as compared to the group of IDD patients. In our IDD cohort, CSF NfL was not significantly higher in the progressive forms of MS as compared to the other clinical phenotypes, as previously reported [32]. This result could have been influenced by the low number of patients with progressive MS (*n* = 8) included in our study. However, we found a nonsignificant increasing trend in CSF NfL values from RIS to CIS, RRMS, and PMS patients, thus suggesting that during the disease course neuro-axonal damage progressively increases.

Nevertheless, within the IDD group, we confirmed the positive correlation between CSF NfL values and several clinical and MRI measures of disease severity. Firstly, CSF NfL values are increased in patients with clinical and/or neuroradiological evidence of disease activity. Indeed, in our study CSF NfL values were significantly higher in patients with a recent relapse, similar to what has been previously reported [18, 33–35]. In addition, we have found that CSF NfL positively correlated with the number of Gd + lesions and was significantly higher in those patients with > 3 Gd + lesions as compared to patients with no Gd + lesions or with 1–3 Gd + lesions. This result is in line with other works showing that both CSF and serum NfL values correlate with an increasing number of Gd + lesions [33, 36–38]. All these findings confirm that in CIS and MS patients NfL may be released from the axons to the interstitial space and then drained in the CSF as the consequence of axonal damage following acute focal inflammation in the CNS. Our results enhance the potential of CSF NfL as a good marker for detecting acute axonal damage in patients with RIS, CIS, and MS [6]. Therefore, the assessment of CSF NfL could participate in the future in defining the “no evidence of disease activity (NEDA)” status in MS patients, as advocated [39].

We have additionally found that in IDD patients CSF NfL values correlate with the degree of disability and with the total number of MRI T2 lesions, as previously reported [32, 33, 38, 40]. As a new finding, moreover, we have shown that CSF NfL values positively correlate with the number of juxtacortical lesions. The correlation between CSF NfL and juxtacortical white matter lesions is of particular interest since juxtacortical lesions are highly specific for MS and they correlate with cortical thinning which, in turn, is a reliable predictor of long-term confirmed disability progression [41, 42]. Of interest, recently it has been shown that MS patients with CSF OCB have higher values of CSF NfL and a higher number of cortical lesions on MRI, thus supporting the hypothesis that cortical and juxtacortical lesions could be associated with a higher degree of axonal damage [43]. Further studies with long-term follow-up times are needed to verify whether CSF NfL can predict long-term disability in MS patients.

### CSF NfL in MCI-AD/AD-dem patients

In our study, CSF NfL concentration was significantly higher in the MCI-AD/AD-dem group as compared with both PD and control subjects, in line with similar results obtained in previous studies [5, 44]. Moreover, the accuracy of CSF NfL in discriminating between MCI-AD/AD-dem patients and controls was high, with an AUC of 0.84 when adjusting for age (the unadjusted AUC was similar at 0.83). In the recent work by Mattsson and colleagues, it was reported that CSF NfL (as measured with the commercially available ELISA kit from UmanDiagnostics) discriminated between AD-dem patients and controls with an AUC of 0.81 when adjusting for the demographics features [44]. Moreover, in the same paper, the AUC for discriminating AD-dem patients and controls adjusted for demographic features was 0.79 for plasma NfL (as measured with the same ELISA kit transferred onto the ultrasensitive single-molecule array platform Simoa), 0.85 for CSF total tau (t-tau), 0.81 for CSF phosphorylated tau (p-tau), and 0.64 for plasma tau [44]. Moreover, the AUC of CSF amyloid beta (Aβ)42, adjusted for both demographic factors and apolipoprotein E (*APOE)* ε4 genotype, was 0.88 [44]. Therefore, our results seem to suggest that the measurement of CSF NfL with the newly developed ELISA has a diagnostic accuracy similar to that of CSF NfL measured with the commercially available ELISA. However, it is worth underlining that the reported high diagnostic accuracy of CSF NfL refers to the discrimination between MCI-AD/AD-dem patients and OND patients. High values of CSF NfL have also been reported in other neurodegenerative disorders that may enter into the differential diagnosis of AD, such as FTD [45]. NfL lacks the pathophysiological specificity of the classical CSF AD biomarkers and, therefore, it is advisable to use it in addition to Aβ42, t-tau, and p-tau in the diagnostic management of AD patients as a general marker of neurodegeneration [44].

Within the MCI-AD/AD-dem group, there was no significant correlation of CSF NfL concentration with MMSE scores at the baseline and at the follow-up or with MMSE score change. This result is in contrast with previous data obtained on larger populations showing a significant correlation between CSF NfL and MMSE performance in AD patients [5]. However, a higher specificity of NfL in detecting cognitive impairment on time-dependent neuropsychological tests (that may more accurately reflect the functionality of larger myelinated axonal connections) has been suggested [44]. Thus, CSF NfL may not be able to significantly reflect the cognitive performance as measured with a screening tool such as MMSE in small cohorts of MCI-AD/AD-dem patients, as in our study.

### CSF NfL in PD patients

Finally, there were no statistically significant differences in CSF NfL concentration between PD and controls, as previously reported in other similar studies performed on CSF NfL [10]. Concordantly, the diagnostic accuracy in discriminating between PD and controls was low with an age-adjusted and unadjusted AUC of 0.69 and 0.62, respectively. CSF NfL has been reported to be significantly higher in neurodegenerative atypical parkinsonism than in PD and, in this context, CSF NfL can help the differential diagnosis with a high sensitivity and specificity [10]. Even if CSF NfL is not significantly increased in PD patients, we found a weak, positive correlation between CSF NfL values and the scores of motor impairment due to PD, as measured with the UPDRS III. Further studies on larger cohorts are required to confirm this result and to assess whether NfL can provide clinically useful prognostic information in the management of PD patients.

## Conclusions

Our findings showed a good analytical performance of a newly developed ELISA for NfL. CSF NfL is confirmed to be a reliable marker of the severity of neuro-axonal damage processes taking place in different neurological diseases. Our results support the use of CSF NfL as a disease intensity marker in MS and AD. Additional studies are required to confirm these results in different cohorts. This achievement could further confirm the role of CSF NfL in predicting long-term outcomes in MS and AD, as well as to confirm its possible prognostic role in PD patients.
